# 3-(4-Methyl­phen­yl)-1-phenyl-3-(4,5,6,7-tetra­hydro-1,2,3-benzoselenadiazol-4-yl)propan-1-one

**DOI:** 10.1107/S160053681102174X

**Published:** 2011-06-18

**Authors:** J. Muthukumaran, M. Nishandhini, S. Chitra, P. Manisankar, Suman Bhattacharya, S. Muthusubramanian, R. Krishna, J. Jeyakanthan

**Affiliations:** a Centre for Bioinformatics, School of Life Sciences, Pondicherry University, Puducherry 605 014, India; b Department of Bioinformatics, Alagappa University, Karaikudi 630 003, India; c Department of Industrial Chemistry, Alagappa University, Karaikudi 630 003, India; d Department of Chemistry, Pondicherry University, Puducherry 605 014, India; e Department of Organic Chemistry, Madurai Kamaraj University, Madurai 625 021, India

## Abstract

In the title compound, C_22_H_22_N_2_OSe, the fused six-membered ring of the 4,5,6,7-tetra­hydro­benzo[*d*][1,2,3] selenadiazole group adopts a near to envelope (*E* form) conformation and the five-membered 1,2,3-selenadiazole ring is essentially planar (r.m.s. deviation = 0.0059 Å). In the crystal, adjacent mol­ecules are inter­linked through weak inter­molecular C—H⋯π inter­actions.

## Related literature

For bond lengths in compounds containing a 1,2,3-selenadiazole group, see: Arsenyan *et al.* (2007 ▶); Saravanan *et al.* (2006*a* ▶,*b* ▶, 2007 ▶, 2008 ▶); Marx *et al.* (2007 ▶, 2008*a* ▶,*b* ▶); Gunasekaran *et al.* (2007*a* ▶,*b* ▶). For biological applications of 1,2,3-selenadiazole derivatives, see: Kuroda *et al.* (2001 ▶); El-Bahaie *et al.* (1990 ▶); El-Kashef *et al.* (1986 ▶); Plano *et al.* (2010 ▶); Padmavathi *et al.* (2002 ▶). For ring puckering analysis, see: Cremer & Pople (1975 ▶). For C—H⋯π inter­actions, see: Desiraju & Steiner (1999 ▶).
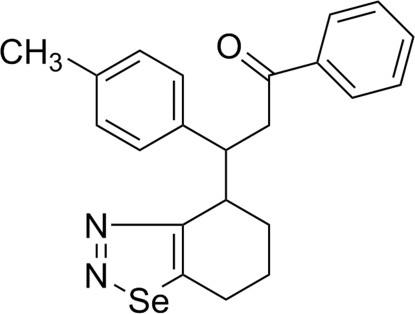


## Experimental

### 

#### Crystal data

C_22_H_22_N_2_OSe
*M* *_r_* = 409.38
Triclinic, 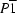

*a* = 8.1485 (9) Å
*b* = 9.7929 (9) Å
*c* = 12.1234 (13) Å
α = 98.707 (9)°
β = 96.387 (9)°
γ = 94.792 (9)°
*V* = 945.36 (17) Å^3^
*Z* = 2
Mo *K*α radiation
μ = 2.00 mm^−1^
*T* = 293 K
0.5 × 0.40 × 0.25 mm

#### Data collection

Oxford Diffraction Xcalibur Eos diffractometer
Absorption correction: multi-scan (*CrysAlis PRO*; Oxford Diffraction, 2009 ▶) *T*_min_ = 0.585, *T*_max_ = 1.000
8343 measured reflections
3339 independent reflections
2615 reflections with *I* > 2σ(*I*)
*R*_int_ = 0.055

#### Refinement

*R*[*F* ^2^ > 2σ(*F* ^2^)] = 0.037
*wR*(*F* ^2^) = 0.100
*S* = 1.00
3339 reflections
236 parameters
H-atom parameters constrained
Δρ_max_ = 0.40 e Å^−3^
Δρ_min_ = −0.66 e Å^−3^

### 

Data collection: *CrysAlis CCD* (Oxford Diffraction, 2009 ▶); cell refinement: *CrysAlis RED* (Oxford Diffraction, 2009 ▶); data reduction: *CrysAlis RED*; program(s) used to solve structure: *SHELXS97* (Sheldrick, 2008 ▶); program(s) used to refine structure: *SHELXL97* (Sheldrick, 2008 ▶); molecular graphics: *ORTEP-3 for Windows* (Farrugia, 1997 ▶) and *PLATON* (Spek, 2009 ▶); software used to prepare material for publication: *PLATON*.

## Supplementary Material

Crystal structure: contains datablock(s) I, New_Global_Publ_Block. DOI: 10.1107/S160053681102174X/zl2374sup1.cif

Structure factors: contains datablock(s) I. DOI: 10.1107/S160053681102174X/zl2374Isup2.hkl

Supplementary material file. DOI: 10.1107/S160053681102174X/zl2374Isup3.cml

Additional supplementary materials:  crystallographic information; 3D view; checkCIF report

## Figures and Tables

**Fig. 1.**
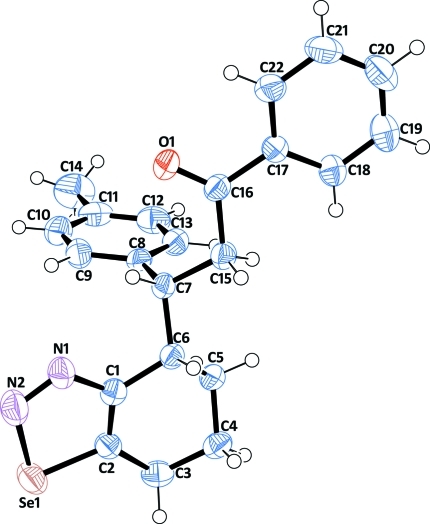
The molecular structure of title compound, showing displacement ellipsoids drawn at the 50% probability level.

**Fig. 2.**
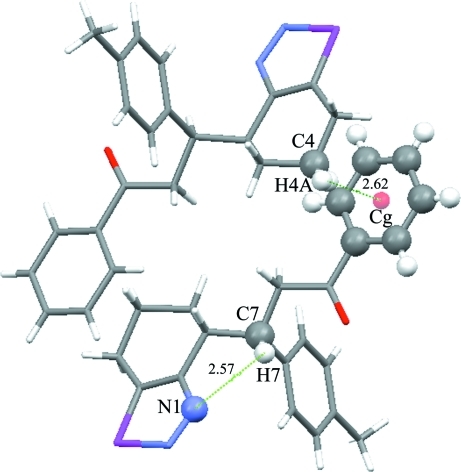
The molecular interaction showing the weak intermolecular C—H···π and intramolecular C-H···N interactions in title compound (Cg is the centroid of C17—C22 ring. Symmetry code for the centroid: 1-x,-y,-z).

**Fig. 3.**
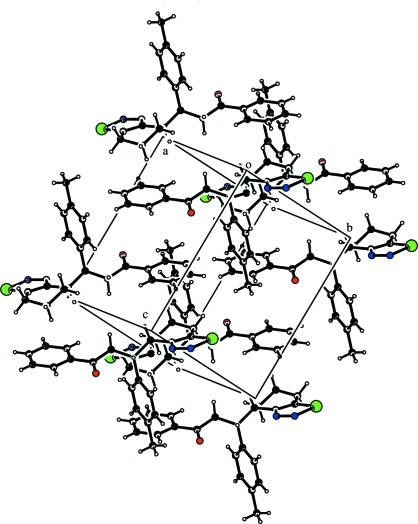
Packing diagram of the title compound.

## supplementary crystallographic information

### Comment

1,2,3-Selenadiazole and its derivatives exhibit various potential biological activities such as anti-fungal (Kuroda *et al.*, 2001), anti-bacterial (El-Kashef *et al.*, 1986), anti-microbial (El-Bahaie *et al.*, 1990), anti-cancer (Plano *et al.*, 2010) and insecticidal (Padmavathi *et al.*, 2002) activities. Considering the importances of the 1,2,3-selenadiazole derivatives, we present herein the single-crystal structure analysis of the title compound. The bond lengths of the 1,2,3-selenadiazole moiety in the title compound are comparable to those observed for selenadiazole moieties in several crystal structures such as 4-methyl-5-ethoxycarbonyl-1,2,3-selenadiazole phenylboronic acid (Arsenyan *et al.*, 2007), diethyl 2-((4-methylphenyl)(4-phenyl-1,2,3-selenadiazol-5-yl)methyl)malonate (Saravanan *et al.*, 2006*a*), 4-(4-chlorophenyl)-5-(1-(4-methoxyphenyl)-2-methyl-2-nitropropyl)-1,2,3-selenadiazole (Saravanan *et al.*, 2006*b*), 3-(4-methylphenyl)-3-(4-(4-methylphenyl)-1,2,3-selenadiazol-5-yl)-2-phenylpropanenitrile (Saravanan *et al.*, 2007), ethyl (Z)-3-(4-chlorophenyl)-2-cyano-3-(4-phenyl-1,2,3-selenadiazol-5-yl)prop-2-enoate (Saravanan *et al.*, 2008), 5-(2-methyl-2-nitro-1-phenylpropyl)-4-phenyl-1,2,3-selenadiazole (Marx *et al.*, 2007), 4-(4-Chlorophenyl)-5-(1-(4-chlorophenyl)-2-methyl-2-nitropropyl)-1,2,3-selenadiazole (Marx *et al.*, 2008*a*), diethyl 2-((4-nitrophenyl)(4-phenyl-1,2,3-selenadiazol-5-yl)methyl)malonate (Marx *et al.*, 2008*b*), 5-[2-methyl-1-(4-methylphenyl)-2-nitropropyl]-4-phenyl-1,2,3-selenadiazole (Gunasekaran *et al.*, 2007*a*) and 4-(4-chlorophenyl)-5-[2-methyl-1-(4-methylphenyl)-2-nitropropyl]-1,2,3-selenadiazole (Gunasekaran *et al.*, 2007*b*). The molecular structure of the title compound is shown in Fig. 1.

The five-membered 1,2,3-selenadiazole moiety (C1/N1/N2/Se1/C2) of the title compound adopts a planar conformation as observed in the selenadiazole moieties of several crystal structures (Arsenyan *et al.*, 2007; Saravanan *et al.*, 2006*a*; Saravanan *et al.*, 2006*b*; Saravanan *et al.*, 2007; Saravanan *et al.*, 2008; Marx *et al.*, 2007; Marx *et al.*, 2008*a*; Marx *et al.*, 2008*b*; Gunasekaran *et al.*, 2007*a*; Gunasekaran *et al.*, 2007*b*). Cremer & Pople puckering analysis (Cremer & Pople, 1975) cannot be performed, for its weighted average absolute torsion angle is 0.89°, which is less than 5.0°. However, the fused six-membered ring (C1/C2/C3/C4/C5/C6) of the 4,5,6,7-tetrahydrobenzo[*d*][1,2,3] selenadiazole group adopts a near envelope (E form) conformation with puckering parameters of Q = 0.485 (3) Å, θ = 47.7 (4)° and Φ = 217.1 (5)°.

The molecular structure is stabilized by an intramolecular C7—H7···N1 interaction (Fig. 2) [C7-N1 distance: 2.96 Å, H7-N1 distance: 2.57 Å and C7-H7···N1 angle 104 °]. The C—H···π interaction (Fig. 2) is observed between C4—H4A···*Cg* (*Cg* is the centroid of the C17—C22 six-membered ring, C···*Cg* distance: 3.549 (3) Å, H-Perp: -2.61 Å), which contributes to the stabilization of crystal packing (Fig. 3, symmetry code for the centroid:  1-x,-y,-z). The bond distance of C—H···π interaction agrees with those described by Desiraju & Steiner (1999).

### Experimental

A mixture of 2-[1-(4-methylphenyl)-3-oxo-3-phenylpropyl]-1-cyclohexanone (1 mmol, 0.32 g) and semicarbazide hydrochloride (1 mmol, 0.11 g) in ethanol (10 ml) was refluxed for 3 h. After completion of the reaction as monitored by TLC, the mixture was poured into ice cold water (50 ml) and the resulting mono-semicarbazone solid was filtered off. Then, a mixture of mono-semicarbazone (1 mmol, 0.38 g) and SeO_2_ (2 mmol, 0.44 g) in tetrahydrofuran (THF) (10 ml) were refluxed on a water bath for 30 minutes. After completion of the reaction as monitored by TLC, the reaction mixture was filtered to remove selenium powder, the filtrate was concentrated under vacuum, and the residue was subjected to column chromatography using a petroleum ether/ethylacetate mixture (95:5; *v*/*v*) as eluent to afford the pure product (Yield: 69%, melting point: 398-399 K). Dissolving the pure compound in a 3:1 mixture of dichloromethane:ethylacetate and slow evaporation of the solvents provided crystals suitable for X-ray analysis. Spectroscopic data for the title compound: IR (KBr): 2940 (C-H), 1679 (C=O), 1585 (N=N), 1351 (C-N)cm^-1^ .

### Refinement

The non-hydrogen atoms were refined anisotropically whereas hydrogen atoms were refined isotropically. The C—H atoms were positioned geometrically (C—H = 0.93–0.98 Å) and were refined using a riding model with *U*_iso_(H) = xU_eq_(C), where *x* = 1.5 for methyl and 1.2 for all other atoms.

### Crystal data

**Table:**

C_22_H_22_N_2_OSe	*Z* = 2
*M**_r_* = 409.38	*F*(000) = 420
Triclinic, *P*1	*D*_x_ = 1.438 Mg m^−^^3^
Hall symbol: -P 1	Mo *K*α radiation, λ = 0.71073 Å
*a* = 8.1485 (9) Å	Cell parameters from 4672 reflections
*b* = 9.7929 (9) Å	θ = 2.9–29.2°
*c* = 12.1234 (13) Å	µ = 2.00 mm^−^^1^
α = 98.707 (9)°	*T* = 293 K
β = 96.387 (9)°	Block, blue
γ = 94.792 (9)°	0.5 × 0.40 × 0.25 mm
*V* = 945.36 (17) Å^3^	

### Data collection

**Table:**

Oxford Diffraction Xcalibur Eos diffractometer	3339 independent reflections
Radiation source: fine-focus sealed tube	2615 reflections with *I* > 2σ(*I*)
graphite	*R*_int_ = 0.055
Detector resolution: 15.9821 pixels mm^-1^	θ_max_ = 25.0°, θ_min_ = 2.9°
ω scans	*h* = −9→9
Absorption correction: multi-scan (*CrysAlis PRO*; Oxford Diffraction, 2009)	*k* = −11→11
*T*_min_ = 0.585, *T*_max_ = 1.000	*l* = −14→14
8343 measured reflections	

### Refinement

**Table:**

Refinement on *F*^2^	Primary atom site location: structure-invariant direct methods
Least-squares matrix: full	Secondary atom site location: difference Fourier map
*R*[*F*^2^ > 2σ(*F*^2^)] = 0.037	Hydrogen site location: inferred from neighbouring sites
*wR*(*F*^2^) = 0.100	H-atom parameters constrained
*S* = 1.00	*w* = 1/[σ^2^(*F*_o_^2^) + (0.050*P*)^2^] where *P* = (*F*_o_^2^ + 2*F*_c_^2^)/3
3339 reflections	(Δ/σ)_max_ = 0.034
236 parameters	Δρ_max_ = 0.40 e Å^−^^3^
0 restraints	Δρ_min_ = −0.66 e Å^−^^3^

### Special details

**Table:**

Geometry. All e.s.d.'s (except the e.s.d. in the dihedral angle between two l.s. planes) are estimated using the full covariance matrix. The cell e.s.d.'s are taken into account individually in the estimation of e.s.d.'s in distances, angles and torsion angles; correlations between e.s.d.'s in cell parameters are only used when they are defined by crystal symmetry. An approximate (isotropic) treatment of cell e.s.d.'s is used for estimating e.s.d.'s involving l.s. planes.
Refinement. Refinement of *F*^2^ against ALL reflections. The weighted *R*-factor *wR* and goodness of fit *S* are based on *F*^2^, conventional *R*-factors *R* are based on *F*, with *F* set to zero for negative *F*^2^. The threshold expression of *F*^2^ > σ(*F*^2^) is used only for calculating *R*-factors(gt) *etc*. and is not relevant to the choice of reflections for refinement. *R*-factors based on *F*^2^ are statistically about twice as large as those based on *F*, and *R*- factors based on ALL data will be even larger.

### Fractional atomic coordinates and isotropic or equivalent isotropic displacement parameters (Å^2^)

**Table:**

	*x*	*y*	*z*	*U*_iso_*/*U*_eq_	
Se1	−0.19156 (4)	0.31704 (3)	−0.12511 (3)	0.05512 (16)	
O1	0.3386 (3)	0.0336 (3)	0.32048 (18)	0.0610 (6)	
N1	−0.0624 (3)	0.1746 (2)	0.0229 (2)	0.0448 (6)	
N2	−0.2056 (3)	0.1974 (3)	−0.0175 (2)	0.0550 (7)	
C1	0.0719 (3)	0.2396 (3)	−0.0161 (2)	0.0343 (6)	
C2	0.0346 (3)	0.3218 (3)	−0.0942 (2)	0.0368 (6)	
C3	0.1612 (4)	0.3990 (3)	−0.1498 (2)	0.0459 (7)	
H3A	0.1697	0.4973	−0.1199	0.055*	
H3B	0.1258	0.3873	−0.2300	0.055*	
C4	0.3299 (3)	0.3453 (3)	−0.1294 (2)	0.0423 (7)	
H4A	0.3314	0.2586	−0.1798	0.051*	
H4B	0.4150	0.4117	−0.1461	0.051*	
C5	0.3690 (3)	0.3219 (3)	−0.0084 (2)	0.0388 (6)	
H5A	0.3656	0.4083	0.0420	0.047*	
H5B	0.4804	0.2942	0.0028	0.047*	
C6	0.2456 (3)	0.2097 (3)	0.0210 (2)	0.0326 (6)	
H6	0.2626	0.1227	−0.0261	0.039*	
C7	0.2761 (3)	0.1843 (3)	0.1445 (2)	0.0327 (6)	
H7	0.1838	0.1185	0.1549	0.039*	
C8	0.2757 (3)	0.3124 (3)	0.2321 (2)	0.0336 (6)	
C9	0.1304 (4)	0.3465 (3)	0.2740 (2)	0.0442 (7)	
H9	0.0321	0.2901	0.2473	0.053*	
C10	0.1276 (4)	0.4621 (3)	0.3547 (2)	0.0503 (8)	
H10	0.0278	0.4816	0.3810	0.060*	
C11	0.2703 (4)	0.5492 (3)	0.3970 (2)	0.0501 (8)	
C12	0.4156 (4)	0.5161 (3)	0.3552 (2)	0.0508 (8)	
H12	0.5137	0.5727	0.3820	0.061*	
C13	0.4181 (3)	0.4012 (3)	0.2750 (2)	0.0415 (7)	
H13	0.5180	0.3823	0.2486	0.050*	
C14	0.2685 (5)	0.6764 (4)	0.4855 (3)	0.0784 (12)	
H14A	0.2668	0.7577	0.4500	0.118*	
H14B	0.3660	0.6855	0.5393	0.118*	
H14C	0.1714	0.6665	0.5229	0.118*	
C15	0.4339 (3)	0.1113 (3)	0.1603 (2)	0.0381 (6)	
H15A	0.4323	0.0374	0.0970	0.046*	
H15B	0.5293	0.1774	0.1599	0.046*	
C16	0.4547 (3)	0.0505 (3)	0.2673 (2)	0.0362 (6)	
C17	0.6188 (3)	0.0024 (2)	0.3039 (2)	0.0328 (6)	
C18	0.7605 (3)	0.0330 (3)	0.2545 (2)	0.0419 (7)	
H18	0.7562	0.0860	0.1969	0.050*	
C19	0.9086 (4)	−0.0160 (3)	0.2917 (3)	0.0579 (8)	
H19	1.0040	0.0053	0.2594	0.069*	
C20	0.9149 (4)	−0.0951 (3)	0.3752 (3)	0.0603 (9)	
H20	1.0144	−0.1281	0.3990	0.072*	
C21	0.7756 (4)	−0.1266 (3)	0.4247 (3)	0.0575 (9)	
H21	0.7810	−0.1804	0.4817	0.069*	
C22	0.6269 (4)	−0.0777 (3)	0.3890 (2)	0.0459 (7)	
H22	0.5325	−0.0987	0.4223	0.055*	

### Atomic displacement parameters (Å^2^)

**Table:**

	*U*^11^	*U*^22^	*U*^33^	*U*^12^	*U*^13^	*U*^23^
Se1	0.0423 (2)	0.0661 (3)	0.0560 (2)	0.01699 (17)	−0.00317 (16)	0.00744 (17)
O1	0.0453 (13)	0.0959 (17)	0.0575 (14)	0.0249 (12)	0.0249 (11)	0.0392 (13)
N1	0.0341 (14)	0.0493 (15)	0.0504 (15)	0.0004 (11)	0.0051 (11)	0.0081 (12)
N2	0.0353 (15)	0.0650 (17)	0.0626 (17)	0.0015 (13)	0.0057 (12)	0.0061 (13)
C1	0.0360 (15)	0.0315 (14)	0.0331 (14)	0.0009 (12)	0.0043 (11)	−0.0008 (11)
C2	0.0379 (16)	0.0372 (15)	0.0332 (14)	0.0080 (12)	0.0015 (12)	−0.0011 (11)
C3	0.0564 (19)	0.0429 (16)	0.0401 (16)	0.0054 (14)	0.0033 (14)	0.0139 (13)
C4	0.0456 (18)	0.0457 (16)	0.0360 (15)	−0.0015 (13)	0.0094 (13)	0.0085 (13)
C5	0.0340 (15)	0.0472 (16)	0.0372 (15)	0.0014 (13)	0.0066 (12)	0.0123 (12)
C6	0.0319 (14)	0.0349 (14)	0.0311 (14)	0.0036 (11)	0.0060 (11)	0.0036 (11)
C7	0.0284 (14)	0.0356 (14)	0.0358 (14)	0.0026 (11)	0.0069 (11)	0.0093 (11)
C8	0.0378 (16)	0.0369 (15)	0.0291 (13)	0.0077 (12)	0.0063 (11)	0.0109 (11)
C9	0.0369 (16)	0.0501 (17)	0.0451 (17)	0.0063 (13)	0.0056 (13)	0.0049 (13)
C10	0.054 (2)	0.0575 (19)	0.0429 (17)	0.0220 (17)	0.0139 (15)	0.0045 (14)
C11	0.075 (2)	0.0421 (17)	0.0343 (16)	0.0160 (17)	0.0026 (15)	0.0064 (13)
C12	0.059 (2)	0.0469 (18)	0.0418 (17)	−0.0043 (15)	−0.0039 (15)	0.0045 (14)
C13	0.0398 (17)	0.0460 (16)	0.0385 (15)	0.0034 (13)	0.0058 (12)	0.0057 (13)
C14	0.110 (3)	0.058 (2)	0.063 (2)	0.023 (2)	0.005 (2)	−0.0098 (18)
C15	0.0382 (16)	0.0398 (15)	0.0397 (15)	0.0099 (13)	0.0106 (12)	0.0094 (12)
C16	0.0387 (16)	0.0383 (15)	0.0329 (14)	0.0055 (12)	0.0092 (12)	0.0054 (11)
C17	0.0357 (15)	0.0295 (13)	0.0316 (14)	0.0050 (11)	0.0031 (11)	−0.0003 (11)
C18	0.0355 (16)	0.0407 (16)	0.0492 (17)	0.0031 (13)	0.0058 (13)	0.0070 (13)
C19	0.0358 (18)	0.063 (2)	0.073 (2)	0.0031 (15)	0.0055 (15)	0.0082 (18)
C20	0.049 (2)	0.066 (2)	0.061 (2)	0.0204 (17)	−0.0099 (17)	0.0025 (17)
C21	0.072 (2)	0.063 (2)	0.0398 (17)	0.0220 (18)	−0.0018 (16)	0.0138 (15)
C22	0.0521 (18)	0.0523 (18)	0.0353 (15)	0.0132 (14)	0.0070 (13)	0.0079 (13)

### Geometric parameters (Å, °)

**Table:**

N1—N2	1.266 (3)	C12—C11	1.383 (4)
N1—C1	1.384 (3)	C12—H12	0.9300
Se1—C2	1.834 (3)	C13—C12	1.375 (4)
Se1—N2	1.887 (3)	C13—C8	1.391 (4)
C1—C2	1.358 (4)	C13—H13	0.9300
C3—C2	1.506 (4)	C14—H14A	0.9600
C3—H3A	0.9700	C14—H14B	0.9600
C3—H3B	0.9700	C14—H14C	0.9600
C4—C5	1.521 (3)	C15—C16	1.506 (3)
C4—C3	1.521 (4)	C15—C7	1.531 (3)
C4—H4A	0.9700	C15—H15A	0.9700
C4—H4B	0.9700	C15—H15B	0.9700
C5—H5A	0.9700	O1—C16	1.216 (3)
C5—H5B	0.9700	C17—C22	1.386 (3)
C6—C1	1.502 (3)	C17—C18	1.390 (4)
C6—C5	1.535 (3)	C17—C16	1.498 (3)
C6—C7	1.552 (3)	C18—C19	1.390 (4)
C6—H6	0.9800	C18—H18	0.9300
C7—C8	1.517 (3)	C19—C20	1.364 (4)
C7—H7	0.9800	C19—H19	0.9300
C9—C8	1.386 (4)	C20—H20	0.9300
C9—C10	1.382 (4)	C21—C20	1.375 (5)
C9—H9	0.9300	C21—H21	0.9300
C10—C11	1.382 (4)	C22—C21	1.389 (4)
C10—H10	0.9300	C22—H22	0.9300
C11—C14	1.518 (4)		
			
N1—N2—Se1	110.78 (19)	C10—C9—H9	119.1
N1—C1—C6	120.6 (2)	C10—C11—C14	121.7 (3)
N2—N1—C1	117.2 (2)	C11—C14—H14A	109.5
C1—C6—C5	109.0 (2)	C11—C14—H14B	109.5
C1—C6—C7	114.5 (2)	C11—C10—C9	121.4 (3)
C1—C6—H6	106.1	C11—C10—H10	119.3
C1—C2—C3	124.5 (2)	C11—C12—H12	119.3
C1—C2—Se1	109.5 (2)	C11—C14—H14C	109.5
C2—Se1—N2	86.70 (11)	C12—C11—C10	117.2 (3)
C2—C1—N1	115.8 (2)	C12—C11—C14	121.0 (3)
C2—C1—C6	123.5 (2)	C12—C13—C8	122.0 (3)
C2—C3—C4	110.6 (2)	C12—C13—H13	119.0
C2—C3—H3A	109.5	C13—C8—C7	122.8 (2)
C2—C3—H3B	109.5	C13—C12—C11	121.3 (3)
C3—C4—H4A	109.3	C13—C12—H12	119.3
C3—C4—H4B	109.3	H14A—C14—H14B	109.5
C3—C2—Se1	125.89 (19)	H14A—C14—H14C	109.5
H3A—C3—H3B	108.1	H14B—C14—H14C	109.5
C4—C5—C6	111.9 (2)	C15—C7—C6	109.07 (19)
C4—C5—H5A	109.2	C15—C7—H7	106.6
C4—C5—H5B	109.2	H15A—C15—H15B	107.7
H4A—C4—H4B	107.9	C16—C15—C7	113.9 (2)
C4—C3—H3A	109.5	C16—C15—H15A	108.8
C4—C3—H3B	109.5	C16—C15—H15B	108.8
C5—C6—C7	114.3 (2)	O1—C16—C17	120.1 (2)
C5—C6—H6	106.1	O1—C16—C15	121.0 (2)
C5—C4—C3	111.7 (2)	C17—C18—H18	120.2
C5—C4—H4A	109.3	C17—C22—H22	119.9
C5—C4—H4B	109.3	C17—C16—C15	118.8 (2)
H5A—C5—H5B	107.9	C18—C17—C16	122.5 (2)
C6—C7—H7	106.6	C18—C19—H19	119.8
C6—C5—H5A	109.2	C19—C18—C17	119.6 (3)
C6—C5—H5B	109.2	C19—C18—H18	120.2
C7—C6—H6	106.1	C19—C20—C21	120.7 (3)
C7—C15—H15A	108.8	C19—C20—H20	119.6
C7—C15—H15B	108.8	C20—C21—C22	119.6 (3)
C8—C9—C10	121.7 (3)	C20—C21—H21	120.2
C8—C9—H9	119.1	C20—C19—C18	120.3 (3)
C8—C13—H13	119.0	C20—C19—H19	119.8
C8—C7—C15	113.0 (2)	C21—C22—C17	120.2 (3)
C8—C7—C6	114.5 (2)	C21—C22—H22	119.9
C8—C7—H7	106.6	C21—C20—H20	119.6
C9—C10—H10	119.3	C22—C17—C18	119.5 (2)
C9—C8—C13	116.4 (3)	C22—C17—C16	118.0 (2)
C9—C8—C7	120.8 (2)	C22—C21—H21	120.2
			
N1—C1—C2—C3	−178.4 (2)	C7—C15—C16—C17	−167.7 (2)
N1—C1—C2—Se1	−0.9 (3)	C8—C13—C12—C11	−0.3 (4)
N2—N1—C1—C2	0.1 (4)	C8—C9—C10—C11	0.1 (4)
N2—N1—C1—C6	−175.4 (2)	C9—C10—C11—C12	0.1 (4)
N2—Se1—C2—C1	1.03 (19)	C9—C10—C11—C14	180.0 (3)
N2—Se1—C2—C3	178.5 (2)	C10—C9—C8—C13	−0.4 (4)
C1—N1—N2—Se1	0.7 (3)	C10—C9—C8—C7	179.4 (2)
C1—C6—C5—C4	−48.7 (3)	C12—C13—C8—C9	0.5 (4)
C1—C6—C7—C8	−70.1 (3)	C12—C13—C8—C7	−179.3 (2)
C1—C6—C7—C15	162.1 (2)	C13—C12—C11—C10	0.0 (4)
C2—Se1—N2—N1	−1.0 (2)	C13—C12—C11—C14	−179.9 (3)
C3—C4—C5—C6	63.0 (3)	C15—C7—C8—C9	−143.5 (2)
C4—C3—C2—C1	14.1 (4)	C15—C7—C8—C13	36.2 (3)
C4—C3—C2—Se1	−162.95 (19)	C16—C17—C18—C19	−179.4 (3)
C5—C6—C1—C2	20.0 (3)	C16—C17—C22—C21	179.0 (3)
C5—C6—C1—N1	−164.9 (2)	C16—C15—C7—C8	65.3 (3)
C5—C6—C7—C8	56.6 (3)	C16—C15—C7—C6	−166.1 (2)
C5—C6—C7—C15	−71.1 (3)	C17—C22—C21—C20	0.1 (4)
C5—C4—C3—C2	−42.7 (3)	C17—C18—C19—C20	0.8 (5)
C6—C1—C2—C3	−3.1 (4)	C18—C19—C20—C21	−0.6 (5)
C6—C1—C2—Se1	174.40 (19)	C18—C17—C16—O1	−172.4 (3)
C6—C7—C8—C9	90.8 (3)	C18—C17—C22—C21	0.1 (4)
C6—C7—C8—C13	−89.5 (3)	C18—C17—C16—C15	11.4 (4)
C7—C6—C5—C4	−178.3 (2)	C22—C17—C18—C19	−0.6 (4)
C7—C6—C1—C2	149.4 (2)	C22—C17—C16—O1	8.8 (4)
C7—C6—C1—N1	−35.5 (3)	C22—C17—C16—C15	−167.4 (2)
C7—C15—C16—O1	16.1 (4)	C22—C21—C20—C19	0.2 (5)
